# The Association between Physical Fitness Performance and Abdominal Obesity Risk among Taiwanese Adults: A Cross-Sectional Study

**DOI:** 10.3390/ijerph17051722

**Published:** 2020-03-06

**Authors:** Po-Fu Lee, Chien-Chang Ho, Nai-Wen Kan, Ding-Peng Yeh, Yun-Chi Chang, Yu-Jui Li, Ching-Yu Tseng, Xin-Yu Hsieh, Chih-Hui Chiu

**Affiliations:** 1 Graduate Institute of Sport Coaching Science, Chinese Culture University, Taipei City 111, Taiwan; f520184fred@yahoo.com.tw; 2 Department of Physical Education, Fu Jen Catholic University, New Taipei City 242, Taiwan; 0024@mail.sa.gov.tw (D.-P.Y.); james76630@hotmail.com (Y.-C.C.); li542058@gmail.com (Y.-J.L.); 015844@mail.fju.edu.tw (C.-Y.T.); r173690@gmail.com (X.-Y.H.); 3 Research and Development Center for Physical Education, Health, and Information Technology, Fu Jen Catholic University, New Taipei City 242, Taiwan; 4 Center for General Education, Taipei Medical University, Taipei City 100, Taiwan; kevinkan@tmu.edu.tw; 5 Department of Physical Therapy and Assistive Technology, National Yang-Ming University, Taipei City 112, Taiwan; 6 Department of Exercise Health Science, National Taiwan University of Sport, Taichung City, Taiwan

**Keywords:** physical fitness performance, abdominal obesity risk, waist circumference, Taiwan

## Abstract

The present study aims to investigate the associations between physical fitness performance and abdominal obesity risk among Taiwanese adults. We conducted a cross-sectional study and reviewed the data derived from the National Physical Fitness Survey in Taiwan (HPFSIT). Data from a total of 62,486 respondents aged 23–64 years were collected in this study. The participants completed a standardized structural questionnaire and a series of anthropometric characteristics (body mass index and waist-to-hip ratio) and physical fitness (3-min step tests, 1-min sit-up tests, and sit-and-reach tests) assessments. Waist circumference was used to define abdominal obesity status. A multiple logistic regression analysis was conducted. Our results presented almost entirely significant associations (except for women) on the 3-min step test. Moreover, the results suggest that muscular strength, endurance, and flexibility may be effective predictors of abdominal obesity among men and women, whereas cardiorespiratory fitness predicted abdominal obesity only in men. According to the results of this study, the fitness–abdominal obesity associations are minor based on a mixed population analysis. However, dose–response relationships have been observed. The present study provides a new perspective by using different types of fitness performance to predict abdominal obesity.

## 1. Introduction

Abdominal obesity negatively impacts human health and occurs when excessive subcutaneous and visceral fat is built up around the stomach and abdomen. Abdominal obesity has been linked to blood lipid disorders, inflammation, insulin resistance or full-blown diabetes [1], and an increased risk of developing cardiovascular disease [2], Alzheimer’s disease, metabolic syndrome [3], and premature mortality [1]. Abdominal obesity is also related to non-alcoholic fatty liver disease (NAFLD), which is the most influential cause of chronic liver disease [4]. Both general obesity and abdominal obesity have become major public health concerns worldwide. The research notes that reducing abdominal fat is one of the largest priorities for avoiding metabolic syndrome [5].

Several anthropometric measures, such as the body mass index (BMI), waist-to-hip ratio (WHR), and waist circumference (WC), have been used to predict metabolic syndrome and abdominal obesity. The accuracy of these methods has been debated. Individually, WC has been considered to be the most accurate indicator to predict abdominal obesity [6] and has been reported to have a valid correlation with metabolic syndrome [7]. Thus, WC can be used to understand a person’s abdominal obesity and can reflect his or her risk of developing metabolic syndrome [8]. Moreover, abdominal fat is associated with physical fitness [9]. For example, numerous studies have indicated that people with higher maximal oxygen uptake usually have better WCs [8,10,11,12]. Recently, Lockie and colleagues [13] highlighted the negative relationships between physical fitness and WC. A greater WC is related to lower performance in push-ups, sit-ups, vertical jumps, the 75-yard pursuit run, and the multistage fitness test (MSFT).

However, studies suggest that one’s individual fitness level significantly attenuates these impacts [14,15,16], although abdominal obesity is generally considered to be a negative factor in health and fitness performance. Moreover, one’s fitness level could be more important than body fat percentage in terms of maintaining general health. For example, research has indicated an association between lower cardiorespiratory fitness and premature mortality in normal weight, overweight, and obese individuals [16]. Thus, body weight and abdominal fat are not the only possible indicators to predict one’s health, as one’s physical fitness level moderates the association between abdominal obesity and health. In other words, a physically fit obese man could be healthier than a physically unfit lean man. Physical fitness level may not predict abdominal obesity as well as abdominal obesity itself. However, to the best of our knowledge, the predictive effects of physical fitness on abdominal obesity have rarely been discussed. Moreover, except for cardiorespiratory fitness, little is known about using muscle strength and flexibility to predict abdominal obesity. The relationship between obesity and fitness is complex and multifactorial relative to one’s health status. Thus, further investigations are needed [17].

Therefore, the purpose of the present study was to investigate the associations between physical fitness performance and abdominal obesity risk from national physical fitness survey data in Taiwan. 

## 2. Material and Methods

### 2.1. Study Design and Participants

A cross-sectional study based on the database of the National Physical Fitness Survey in Taiwan (NPFSIT) was conducted. The NPFSIT is governed by the Sports Administration, Ministry of Education in Taiwan. All data from the NPFSIT database are anonymized and have been released for public research purposes [18]. Participants were recruited from a total of 46 physical fitness test stations among 22 cities and counties in Taiwan from October 2014 to March 2015. Informed consent was obtained from participants after a full explanation of the survey. Moreover, the design and procedure of data analysis in this study were approved by the Institutional Review Board (IRB) of Fu Jen Catholic University in Taiwan (FJU-IRB C108006). In total, 62,486 participants (29,585 men and 32,901 women) aged 23–64 years were included in this analysis.

### 2.2. Data Collection Procedures

A standardized structural questionnaire was applied for the face-to-face interviews prior to the physical fitness tests. Data on the participants’ socio-demographic characteristics (age, gender, education, monthly income, and marital status), lifestyle (smoking, betel-nut chewing, and diet), and perceived health status were collected. Educational status was divided into elementary school or lower, junior or senior school, and college or higher; monthly income was divided into ≦20,000 NTD (New Taiwan Dollar), 20,001–40,000 NTD, and ≧40,001 NTD; and marital status was divided into married, never married, and divorced/separation/widowed. Smoking and betel-nut chewing habits are listed as follows: never users, former users, and current users. Perceived health status is divided into excellent or good; fair; and very bad or poor.

After the questionnaire, each participant was asked to measure his or her resting heart rate and blood pressure for the preliminary safety screen before conducting the physical fitness tests. Participants who had systolic blood pressure over 140 mmHg and/or diastolic blood pressure over 90 mmHg, or who were suffering from heart disease, hypertension, chest pain, vertigo, and musculoskeletal disorders, were excluded. The anthropometric variables and physical fitness measurements of each participant were measured after the abovementioned procedures were completed.

### 2.3. Anthropometrics and Body Composition

Anthropometric variables were measured using bodyweight, height, WC, and hip circumference (HC). The WC measurements (measured to the nearest 0.1 cm) were performed twice at the midway between the lowest rib and iliac crest after a normal exhalation, and the mean value was used. The HC measurements (measured to the nearest 0.1 cm) were performed twice at the site of the largest convexity of the buttocks below the hip plates, and the mean value was used.

Body composition was assessed using the BMI and WHR as the primary references in this study. The BMI was calculated as the body weight (kg) divided by the square of the height (m^2^). The WHR was calculated as WC (cm) divided by HC (cm). Then, the participants were classified into an abdominally obese group and a non-abdominally obese group. The WC cutoff points were suggested by the Health Promotion Administration, Ministry of Health and Welfare in Taiwan. WC values over 90 cm in men and 80 cm in women indicate abdominal obesity [19].

### 2.4. Physical Fitness Assessment

The data of the following physical fitness measurements were collected: Cardiorespiratory endurance by a 3-min step test [20], muscle strength and endurance by a 1-min sit-up test (reps/ min) [21], and flexibility by a sit-and-reach test (cm) test [22]. Except for the sit-and-reach test, all of the tests were performed once. The best performance of the two sit-and-reach tests was applied. The assessments were conducted by well-trained examiners who were certified by regional training programs and had been previously published [18,20].

Participants were asked to avoid any other physical activity before the tests. A 10-min warmup was performed and introduced by the examiner prior to the physical fitness assessment. All participants were performed about the tests in the following order with a sufficient break period (3–5 min) between tests: the 1-min sit-up test, 2-min step test, the sit-and-reach test, and the 3-min step test.

### 2.5. Statistical Analysis

Data were analyzed using SAS 9.4 (SAS Institute, Cary, NC, USA). Student’s *t*-test was applied to analyze continuous variables, and a chi-square test was used for the analysis of the categorical variables. A multiple logistic regression analysis was used to evaluate the association between cardiorespiratory endurance, muscle strength, endurance or flexibility, and abdominal obesity. All regression models were adjusted for age, BMI, education, monthly income, marital status, self-reported health status, smoking status, and chewing betel nuts. Age-adjusted odds ratios (ORs) and 95% confidence intervals (CIs) were calculated. In order to examine the dose–response relationship between physical fitness and abdominal obesity, four different categories (quartiles) were created for each physical fitness measurement according to gender. The lowest quartile was composed of participants who had the worst results in each physical fitness measurement, while the highest quartile was composed of participants who had the best results in each physical fitness measurement. Values are presented as the means ± standard deviation (SD) or as frequency percentages. Statistical results are considered significant at *p* < 0.05.

## 3. Results

In total, 29,585 men and 32,901 women were included in this study. Table 1 lists the characteristics of the study participants classified by their abdominal obesity status. A total of 8334 men and 10,084 women were abdominally obese, while 21,351 men and 22,817 women were non-abdominally obese. Both genders in the abdominally obese group were older and had higher BMI, WC, HC, and WHR values. Significant differences were found in education, income, marital status, self-reported health status, smoking status, and betel-nut chewing status among both the men and women.

Table 2 indicates that the non-abdominally obese group had significantly higher results for all physical fitness tests among both men and women. These results are generally consistent with those of previous studies [8,10‒13]. Table 3 presents the odds ratios (ORs) of each physical fitness measurement in relation to abdominal obesity after adjustment for potential confounders. The association between the 3-min step test results and abdominal obesity disappeared in women (OR, 1.00; 95% confidence interval [CI] 1.00–1.00; *p* = 0.193). The other values remained the same. 

Table 4 presented the multivariate-adjusted ORs for abdominal obesity in relation to the quartiles of health-related physical fitness measurements after adjustment for potential confounders. Using a 3-min step test result of >63.38 as the baseline, participants with 3-min step test results <50.56 exhibited the highest risk of abdominal obesity (OR, 1.45; 95% CI 1.30–1.62) among men. However, there was no significant relationship between the 3-min step test results and abdominal obesity in women when using >62.07 as the baseline in Model 2. When a 1-min sit-up test score of >35.00 was set as the baseline, male participants with 1-min sit-up test scores of <22.00 had the highest risk of abdominal obesity (OR, 2.23; 95% CI 1.94–2.55) after adjustment. However, for women, scores between 19.00 and 25.00 did not present a significant relationship between the 1-min sit-up test results and a risk of abdominal obesity (OR, 1.05; 95% CI 0.95–1.17) when a 1-min sit-up test score of >25.00 was used as the baseline. Using a sit-and-reach test score of >29.00 as the baseline, male participants with scores of <14.00 had the highest risk of abdominal obesity (OR, 1.86; 95% CI 1.66–2.09) after adjustment. For women, when a sit-and-reach test score of >35.00 was set as the baseline, participants with scores of <20.00 had the highest risk of abdominal obesity (OR, 1.58; 95% CI 1.43–1.76) after adjustment.

## 4. Discussion

The purpose of the present study was to investigate the associations between physical fitness performance and abdominal obesity risk. A large nationwide cross-sectional study was conducted using a representative database. Based on the mixed populations, minor associations were observed in almost all of the physical fitness and abdominal obesity measurements among both genders, except for the 3-min step test for women. However, according to the results of the physical fitness level, it is suggested that, in addition to cardiorespiratory fitness, muscle strength/endurance and flexibility could also be good predictors of abdominal obesity. Specifically, participants who performed in the worst quartile in the 1-min sit-up test may increase their risk of abdominal obesity by around 1.6 to 2.2 times. Further, the analysis of the sit-and-reach test presented the same results (OR for men, 1.86; OR for women, 1.58). Male populations were influenced the most.

Regular physical activity has been reported to cause an energy imbalance, which reduces BMI and body fat, abdominal fat in particular. Among individuals of a constant body weight, regular exercise effectively reduces visceral fat and abdominal obesity levels [23]. Moreover, people with satisfactory cardiorespiratory fitness have been reported to possess high adiponectin and leptin concentrations [24], low amounts of abdominal subcutaneous fat and visceral fat [25], and reduced inflammation and insulin resistance [26]. Satisfactory cardiorespiratory fitness was also reported to reduce the likelihood of abdominal obesity effectively [10]. The results of the present study concur with the aforementioned findings, indicating that the 3-min step test effectively predicted abdominal obesity, particularly among men.

However, after confounder adjustment, cardiorespiratory fitness was revealed not to effectively predict abdominal obesity in women. This is possibly because this study employed a 3-min step test rather than a test of maximal oxygen uptake. Testing maximal oxygen uptake is time-consuming and requires high exercise intensity. Therefore, it is not necessarily suitable for everyone. In contrast, a 3-min step test is relatively efficient and can be practiced by most people. The application of different styles of tests on cardiorespiratory fitness for different populations should be further investigated. On the other hand, studies have reported that women exhibit higher adiponectin concentrations than men [27]. Adiponectin has been indicated to affect visceral fat and abdominal obesity levels [24]. The hormonal differences between women and men have also been reported to cause differences in where fat is stored and, thus, differences in the amount of abdominal fat [28]. In this study, however, no data appeared to confirm why cardiorespiratory fitness did not effectively predict abdominal obesity in women. Future studies may explore the association of sex with abdominal obesity in addition to its association with cardiorespiratory fitness, as well as use more evidential parameters in their study design.

The results of this study demonstrated that muscular strength and endurance are crucial indicators of abdominal obesity. Studies have shown that muscular endurance training effectively reduces the WC of people with obesity [29]. Compared with exercises involving only muscular endurance, those involving both muscular strength and endurance are more effective for reducing WC in adults [30]. One study involving 846 young men as participants revealed a significant correlation between the 1-min sit-up test results and both WC and the amount of body fat [31]. However, no studies have covered a wide range of age groups in their data analyses or explored whether muscular strength and endurance test results effectively predict abdominal obesity after excluding potential confounders, as the present study did. As demonstrated in the present study, the 1-min sit-up test results effectively predicted abdominal obesity levels in both men and women.

Furthermore, muscular flexibility was confirmed to predict abdominal obesity in both male and female participants effectively. Studies have reported that exercise effectively reduces the risk of cardiovascular diseases [32]. However, because of a rise in both the participants’ muscular strength and body balance, the reduced risk of cardiovascular diseases could not necessarily be attributed to muscular flexibility. Furthermore, no studies have been conducted on the relationship between muscular flexibility and abdominal obesity. Generally, people who engage in physical activity regularly exhibit favorable cardiorespiratory fitness and muscular strength, endurance, and flexibility [33]. Further research may determine whether muscular flexibility alone can serve as a crucial indicator of abdominal obesity.

The strength of the present study is its use of representative data for analysis. However, some limitations should be addressed. First, the research population was composed of Taiwanese adults aged 23 to 64 years. Future studies should be conducted on participants of different ages, different races, and different cultures (lifestyles). Second, although WC has been reported to be a reliable indicator for abdominal obesity [7‒9,11‒14], the discrimination between abdominal obesity and general obesity cannot be guaranteed. It is also limiting to use these measurements to predict subcutaneous and visceral fat in terms of fitness–abdominal associations. Future studies should carefully address this factor or use specific techniques, such as abdominal ultrasonography, bioelectrical impedance analysis (BIA), dual energy X-ray absorptiometry (DXA), or biochemical indicator experiments. Third, some confounders, such as alcohol consumption, food intake, and dietary history, have not been investigated in the NPFSIT. These factors are essential for physical fitness performance, as well as abdominal obesity status. In the future, NPFSITs should include these important factors to maximize the value of the NPFSIT database. Last but not least, the present study adopted a cross-sectional study design to determine the predictive effect of different types of physical fitness performance on abdominal obesity. Although we have clearly defined the relationship between the independent and dependent variables in our analysis, no cause-and-effect relationship can be guaranteed. We suggest future studies be conducted with a longitudinal study design to produce a more complete understanding of this matter. 

## 5. Conclusions

According to the results of this study, fitness–abdominal obesity associations are insignificant based on a mixed population analysis. However, dose–response relationships have been observed. Physical fitness levels were moderately effective at predicting abdominal obesity. Specifically, different levels of performance in muscular strength, endurance, and flexibility effectively predicted abdominal obesity in both genders, whereas cardiorespiratory fitness predicted abdominal obesity only in men. However, the mechanism by which physical fitness affects abdominal obesity remains unclear. Further investigations are still needed.

## Figures and Tables

**Table 1:** Characteristics of the study participants with or without abdominal obesity among Taiwanese adults.

Variables	Men (*N* = 29,585)			Women (*N* = 32,901)		
	Abdominal Obesity (*n* = 8334)	Non-Abdominal Obesity (*n* = 21,351)	*p*	Abdominal Obesity (*n* = 10,084)	Non-Abdominal Obesity (*n* = 22,817)	*p*
Age (years)	42.76 ± 11.23	39.61 ± 11.62	<0.001 *	46.45 ± 11.62	41.00 ± 11.52	<0.001 *
Body weight (kg)	82.56 ± 8.40	68.15 ± 8.21	<0.001 *	65.41 ± 8.68	53.88 ± 6.16	<0.001 *
Height (cm)	171.52 ± 6.19	170.36 ± 6.36	<0.001 *	158.35 ± 6.02	158.34 ± 5.66	0.944
BMI (kg/m^2^)	28.07 ± 2.56	23.48 ± 2.48	<0.001 *	26.10 ± 3.27	21.50 ± 2.32	<0.001 *
WC (cm)	95.15 ± 4.55	80.38 ± 5.99	<0.001 *	86.48 ± 5.90	71.16 ± 5.14	<0.001 *
HC (cm)	102.95 ± 4.86	94.56 ± 5.01	<0.001 *	99.94 ± 5.95	91.62 ± 5.09	<0.001 *
WHR	0.93 ± 0.04	0.85 ± 0.05	<0.001 *	0.87 ± 0.05	0.78 ± 0.05	<0.001 *
Education level (%)			<0.001 *			<0.001 *
Elementary school or lower	2.49	1.44		9.27	3.59	
Junior or senior school	26.86	23.08		38.98	27.89	
College or higher	70.64	75.48		51.76	68.51	
Income level (%)			<0.001 *			<0.001 *
≦20,000 NTD	13.66	15.52		32.96	23.62	
20,001–40,000 NTD	33.28	35.10		42.93	48.86	
≧40,001 NTD	53.06	49.38		24.11	27.52	
Marital status (%)			<0.001 *			<0.001 *
Never married	54.88	52.06		58.23	54.10	
Married	42.13	45.88		34.86	41.63	
Divorced/separation/widowed	3.00	2.06		6.90	4.28	
Self-reported health status (%)			<0.001 *			<0.001 *
Excellent or good	55.53	64.72		56.23	61.00	
Fair	35.39	30.05		35.46	32.78	
Very bad or poor	9.08	5.23		8.31	6.22	
Smoking status (%)			<0.001 *			<0.001 *
Never	64.81	72.72		94.79	96.15	
Current	23.13	18.21		3.73	2.73	
Former	12.06	9.07		1.48	1.12	
Chewing betel nuts			<0.001 *			<0.001 *
Never	87.05	91.97		98.02	99.40	
Current	5.30	2.76		1.61	0.40	
Former	7.64	5.27		0.37	0.21	

Abbreviations: BMI, body mass index; HC, hip circumference; NTD, New Taiwan Dollar; SD, standard deviation; WC, waist circumference; WHR, waist-to-hip ratio.; values are expressed as the means ± standard deviation (SD) and %.; * student *t*-test or one-way ANOVA, *p* < 0.05.

**Table 2:** Health-related physical fitness measurements based on abdominal obesity status among Taiwanese adults.

Variables	Men (*N* = 29,585)			Women (*N* = 32,901)		
	Abdominal Obesity (*n* = 8,334)	Non-Abdominal Obesity (*n* =21,351)	*p*	Abdominal Obesity (*n* = 10,084)	Non-Abdominal Obesity (*n* = 22,817)	*p*
3-min step test	55.61 ± 10.13	58.52 ± 10.54	<0.001 *	53.93 ± 12.59	56.80 ± 11.02	<0.001 *
1-min sit-up test (reps/min)	25.57 ± 9.77	29.80 ± 10.14	<0.001 *	14.22 ± 10.19	19.39 ± 10.25	<0.001 *
Sit-and-reach test (cm)	19.63 ± 10.29	22.28 ± 10.68	<0.001 *	26.27 ± 10.68	28.00 ± 11.17	<0.001 *

Abbreviations: SD, standard deviation; WC, waist circumference. Values are expressed as the means ± SD. * student *t* test, *p* <0.05.

**Table 3:** Multivariate adjusted ORs for abdominal obesity in relation to health-related physical fitness measurements after adjustment for potential confounders.

Variables	Model 1 (Unadjusted)			Model 2 (Adjusted)		
	OR	95% CI	*p*	OR	95% CI	*p*
Men						
3-min step test	0.97	0.97–0.98	<0.001 *	0.99	0.99–0.99	<0.001 *
1-min sit-up test (reps/min)	0.96	0.96–0.97	<0.001 *	0.97	0.97–0.98	<0.001 *
Sit-and-reach test (cm)	0.99	0.98–0.99	<0.001 *	0.98	0.97–0.98	<0.001 *
Women						
3-min step test	0.98	0.98–0.98	<0.001 *	1.00	1.00–1.00	0.193
1-min sit-up test (reps/min)	0.95	0.95–0.96	<0.001 *	0.98	0.98–0.99	<0.001 *
Sit-and-reach test (cm)	1.00	0.99–1.00	0.001 *	0.98	0.98–0.99	<0.001 *

Abbreviations: BMI, body mass index; CI, confidence interval; OR, odds ratio. The logistic regression models adjusted for age, BMI, education, monthly income, marital status, self-reported health status, smoking status, and chewing betel nuts; * logistic regression, *p* < 0.05.

**Table 4:** Multivariate adjusted ORs for abdominal obesity in relation to the quartiles of health-related physical fitness measurements after adjustment for potential confounders.

Variables	Model 1 (Unadjusted)			Model 2 (Adjusted)		
	OR	95% CI	*p*	OR	95% CI	*p*
Men						
3-min step test						
<50.56	2.07	1.92–2.23	<0.001 *	1.45	1.30–1.62	<0.001 *
50.56–56.24	1.52	1.41–1.64	<0.001 *	1.21	1.08–1.35	0.001 *
56.25–63.38	1.27	1.18–1.38	<0.001 *	1.22	1.09–1.36	<0.001 *
>63.38	Ref.	—	—	Ref.	—	—
Test for trend	*p* < 0.001 *			*p* < 0.001 *		
1–min sit–up test (reps/min)						
<22.00	3.10	2.86–3.36	<0.001 *	2.23	1.94–2.55	<0.001 *
22.00–28.99	2.24	2.06–2.42	<0.001 *	1.79	1.58–2.02	<0.001 *
29.00–35.00	1.71	1.58–1.86	<0.001 *	1.49	1.33–1.67	<0.001 *
>35.00	Ref.	—	—	Ref.	—	—
Test for trend	*p* < 0.001 *			*p* < 0.001 *		
Sit–and–reach test (cm)						
<14.00	1.49	1.38–1.61	<0.001 *	1.86	1.66–2.09	<0.001 *
14.00–20.99	1.40	1.30–1.52	<0.001 *	1.45	1.30–1.63	<0.001 *
21.00–29.00	1.27	1.18–1.37	<0.001 *	1.30	1.17–1.45	<0.001 *
>29.00	Ref.	—	—	Ref.	—	—
Test for trend	*p* < 0.001 *			*p* < 0.001 *		
Women						
3–min step test						
<49.45	1.75	1.64–1.88	<0.001 *	1.05	0.96–1.16	0.294
49.45–54.87	1.24	1.15–1.33	<0.001 *	1.08	0.98–1.30	0.113
54.88–62.07	1.09	1.01–1.17	0.019 *	1.00	0.91–1.10	0.977
>62.07	Ref.	—	—	Ref.	—	—
Test for trend	*p* < 0.001 *			*p* = 0.139		
1–min sit–up test (reps/min)						
<10.00	3.95	3.65–4.27	<0.001 *	1.58	1.40–1.79	<0.001 *
10.00–18.99	2.34	2.17–2.52	<0.001 *	1.25	1.12–1.40	<0.001 *
19.00–25.00	1.54	1.42–1.66	<0.001 *	1.05	0.95–1.17	0.352
>25.00	Ref.	—	—	Ref.	—	—
Test for trend	*p* < 0.001 *			*p* < 0.001 *		
Sit–and–reach test (cm)						
<20.00	1.15	1.07–1.24	<0.001 *	1.58	1.43–1.76	<0.001 *
20.00–27.99	1.25	1.16–1.34	<0.001 *	1.41	1.28–1.56	<0.001 *
28.00–35.00	1.31	1.22–1.41	<0.001 *	1.31	1.19–1.44	<0.001 *
>35.00	Ref.	—	—	Ref.	—	—
Test for trend	*p* = 0.005 *			*p* < 0.001 *		

Abbreviations: BMI, body mass index; CI, confidence interval; OR, odds ratio. The logistic regression models were adjusted for age, BMI, education, monthly income, marital status, self-reported health status, smoking status, and chewing betel nuts. * logistic regression, *p* < 0.05.

## Abbreviations

The following abbreviations are used in this manuscript:

BMI	body mass index
CI	confidence interval
HC	hip circumference
IRB	Institutional Review Board
NPFSIT	National Physical Fitness Survey in Taiwan
NTD	New Taiwan Dollar
OR	odds ratio
SD	standard deviation
SAS	statistical analysis system
WC	waist circumference
WHR	waist-to-hip ratio
